# Association of Serum Bile Acid Profile with Diet and Physical Activity Habits in Japanese Middle-Aged Men

**DOI:** 10.3390/nu16193381

**Published:** 2024-10-04

**Authors:** Wataru Aoi, Teruhide Koyama, Akira Honda, Tomohisa Takagi, Yuji Naito

**Affiliations:** 1 Laboratory of Nutrition Science, Graduate School of Life and Environmental Sciences, Kyoto Prefectural University, Kyoto 6068522, Japan; 2 Department of Epidemiology for Community Health and Medicine, Kyoto Prefectural University of Medicine, Kyoto 6028566, Japan; 3 Division of Gastroenterology and Hepatology, Tokyo Medical University Ibaraki Medical Center, Ibaraki 3000395, Japan; akihonda@tokyo-med.ac.jp; 4 Molecular Gastroenterology and Hepatology, Graduate School of Medical Science, Kyoto Prefectural University of Medicine, Kyoto 6028566, Japan; 5 Department for Medical Innovation and Translational Medical Science, Graduate School of Medical Science, Kyoto Prefectural University of Medicine, Kyoto 6028566, Japan; 6 Department of Human Immunology and Nutrition Science, Graduate School of Medical Science, Kyoto Prefectural University of Medicine, Kyoto 6028566, Japan

**Keywords:** bile acids, deconjugation, lifestyle, green tea, physical activity

## Abstract

Background/Objectives: Circulating bile acid (BA) profiles change with lifestyle and are closely related to intestinal BA metabolisms such as deconjugation and conversion to secondary BAs. The composition of BA in the blood is involved in systemic nutrient metabolism and intestinal health. Herein, we explored the associations of lifestyle and physical fitness with the circulating BA profile of middle-aged men. Methods: Data of 147 male participants (aged 50–64 years; BMI < 26 kg/m^2^; no medication for diabetes or dyslipidemia) from the Japan Multi-Institutional Collaborative Cohort study were analyzed. Serum concentrations of 15 types of BAs were examined for associations with variables on dietary habits, physical-activity habits, and physical fitness. Results: Green tea intake was positively associated with the deconjugation ratio of total BAs (*p* = 0.028) and negatively associated with secondary BA levels (free deoxycholic acid [DCA] (*p* = 0.078), glyco-DCA (*p* = 0.048), and tauro-DCA (*p* = 0.037)). In contrast, physical activity was negatively associated with the deconjugation ratio (*p* = 0.029) and secondary BA levels (free DCA (*p* = 0.098), and free lithocholic acid (*p* = 0.009)). Grip strength was also negatively associated with secondary BA levels (tauro-DCA (*p* = 0.041)) but was not associated with the deconjugation ratio. Energy and fat intake and skeletal muscle mass were not associated with the deconjugation ratio or secondary BA levels. Conclusions: The study findings suggest that lifestyle-associated changes in serum deconjugated and secondary BAs indicate improvements in nutrient metabolism and the intestinal environment.

## 1. Introduction

Lifestyle is closely associated with the risk and morbidity of metabolic diseases such as obesity, type 2 diabetes, and dyslipidemia. Dietary habits are major lifestyle factors that affect metabolic homeostasis. Energy intake and protein–fat–carbohydrate balance modulate body composition, such as fat and skeletal muscle mass, and the metabolic capacity of nutrients. Micronutrients, such as vitamins and minerals, are involved in the cellular metabolic reactions of protein, fat, and carbohydrates. Other phytochemicals such as polyphenols and carotenoids contained in natural foods can also contribute to metabolic homeostasis by regulating energy and protein metabolism [1,2]. Specifically, catechins, quercetin, and astaxanthin have been reported to improve mitochondria biogenesis and insulin sensitivity [3,4,5]. In addition, daily physical activity is another lifestyle-related factor that regulates metabolic homeostasis. Physical activity increases energy consumption with the metabolism of carbohydrates and fat, as well as the synthesis of body protein, which adaptively leads to changes in fat and skeletal muscle mass as well as parameters of physical fitness such as strength and endurance. Accumulating evidence has shown that various molecular events in response to diet and physical activity are involved in improved insulin sensitivity, mitochondrial respiration, lipolysis, and protein anabolism in the skeletal muscle, liver, and adipose tissues [6,7]. Various metabolic regulatory factors derived from these tissues and intestinal microbiota have been suggested to influence each other through crosstalk [8,9].

Bile acid (BA) has been shown to act as a metabolic regulator. Primary BAs such as cholic acid (CA) and chenodeoxycholic acid (CDCA) are generated and conjugated with glycine or taurine to form glyco-CA, glyco-CDCA, tauro-CA, and tauro-CDCA in the liver. These primary BAs are exported into bile and metabolized by various enzymes of specific bacteria in the intestine [10]. Glycine and taurine in the conjugated BAs are deconjugated, and CA and CDCA are metabolized by multi-step 7α-dehydroxylation of bacteria to form the secondary BAs (deoxycholic acid [DCA] and lithocholic acid [LCA]). Approximately 95% of intestinal BAs are absorbed into the blood through the portal vein and taken into the liver again or other organs. Accumulating evidence suggests that circulating BAs can regulate nutrient metabolism by activating BA-specific receptors, the farnesoid X receptor (FXR), and the transmembrane G protein-coupled receptor (TGR)-5 in metabolic tissues [11]. Furthermore, plasma-conjugated and free-form BAs show different metabolic effects in skeletal muscles and adipose tissues [12,13]. Additionally, secondary BAs have been known to affect intestinal health and diseases such as abnormal intestinal permeability and colon tumorigenesis [14]. The circulating secondary BA level has been suggested to indicate intestinal conditions [15].

The composition of circulating BAs is changed by diet and physical activity in mice, which may mediate their metabolic effects [12,16]. Alterations in gut microbiota can affect BA metabolism and alter the circulating BA profile. Given that diet and exercise regulate systemic metabolism, the lifestyle–gut microbiota–BA axis functions as a metabolic regulatory system. Recently, we demonstrated that gut microbiota transplantation in mice altered circulating BA composition and glucose metabolism. Thus, these changes in BA composition may be involved in metabolic improvement and intestinal health associated with physical activity and diet. However, the association between lifestyle habits and plasma BA profiles in healthy humans has not yet been clarified. Middle-aged Japanese men are particularly known to have a high proportion of risk factors for metabolic disorders that can lead to type 2 diabetes and cardiovascular disorders [17]. Since such metabolic disorders are mainly caused by lifestyle, investigating the relationship between lifestyle and circulating BAs is important. In this study, we explored diet, physical activity, and fitness levels that are associated with the BA profile in the peripheral blood of middle-aged Japanese men.

## 2. Methods

### 2.1. Participants and Experimental Design 

This cross-sectional study included individuals who were enrolled in the second survey of the Japan Multi-Institutional Collaborative Cohort (J-MICC) Study in the Kyoto area from 2013 to 2017 [18]. The participants underwent measurements of height and body weight, provided blood samples, and completed self-reported questionnaires. From 3911 participants, 147 men (aged 50–64 years), belonging to an age group with a high proportion of risk factors for metabolic disorders, were randomly selected (Figure S1), and their serum samples were analyzed for serum BA levels. To minimize the effects of body fat, medications, and alcohol intake on BAs, the exclusion criteria were as follows: body mass index (BMI) > 26 kg/m^2^, alcohol consumption > 3 cups/day, and use of medications related to diabetes and dyslipidemia. Notably, to ensure the age-limited sample size for analysis, participants with BMI up to 26 kg/m^2^ were included. This study was approved by the Institutional Ethics Committees of the Kyoto Prefectural University (No. 199) and Kyoto Prefectural University of Medicine (No. ERB-C-1947) and conducted in accordance with the principles of the Declaration of Helsinki. All participants provided written informed consent before participation.

### 2.2. Serum BA Analysis

Serum BA profiles were analyzed by a liquid chromatography-tandem mass spectrometry (LC-MS/MS) system [12]. Briefly, serum was diluted 100-fold with ^2^H-labeled internal standards and 0.5 M potassium phosphate buffer (pH 7.4). The mixture was applied to a Bond Elut C18 cartridge (200 mg; Agilent Technologies, Santa Clara, CA, USA). The target molecules were eluted using water/ethanol (1:9, *v*/*v*). The eluate was evaporated under nitrogen until dryness and dissolved in 20 mM ammonium acetate buffer (pH 7.5)/methanol (1:1, *v*/*v*). An aliquot of each sample was injected into the LC-MS/MS system for analysis. Chromatographic separation was performed using a Hypersil GOLD column (Thermo Fisher Scientific, Waltham, MA). A mixture of 20 mM ammonium acetate buffer (pH 7.5), acetonitrile, and methanol (70:15:15, *v*/*v*) was used as the initial mobile phase, which was gradually changed to 30:35:35 (*v*/*v*/*v*) over 20 min. A total of 15 BA levels, including glycine- and taurine-conjugated or deconjugated (free) forms for primary (CA and CDCA) and secondary (DCA, LCA, and ursodeoxycholic acid [UDCA]) BAs, were measured. Negative electrospray ionization-selected reaction monitoring and high-performance liquid chromatography data for each BA are shown in Table S1. Using the values of each BA, the deconjugation form/(deconjugation + conjugation forms) ratio was calculated to obtain the ratio of deconjugated BAs to examine BA conversion between the free and conjugated forms.

### 2.3. Data Collection and Measurements

This study evaluated lifestyle and clinical variables (smoking and alcohol consumption status and physical activity) using self-administered questionnaires. Information regarding green tea and coffee consumption was obtained in terms of frequency and intake and categorized as never, 1–3 times/month, 1–2 times/week, 3–4 times/week, 5–6 times/week, 1 time/day, 2 times/day, or ≥3 times/day. A validated short food frequency questionnaire (FFQ) was used to estimate daily nutrient intake [19], and the frequency of consumption of 46 foods and beverages in the year prior to the J-MICC study was recorded. Among the 46 foods, the frequency of consumption of staple foods (rice, bread, and noodles) at breakfast, lunch, and dinner was grouped into six categories from rarely to every day. For the remaining 43 foods and beverages, the frequency of consumption was grouped into eight categories from rarely to ≥3 times per day. The alcoholic content of each type of beverage was calculated, and alcohol consumption was determined by calculating the number of drinks per day, which was subsequently converted into the Japanese sake unit, “gou” (180 mL), equivalent to 23 g of ethanol. To assess medication use, participants were asked whether they were taking medications for hypertension, dyslipidemia, or diabetes at least once a week.

Physical activity was determined using a format similar to that of the International Physical Activity Questionnaire (IPAQ) [20]. Leisure-time physical activity was measured in terms of metabolic equivalents (METs), as previously described [21]. In brief, MET-hours per day of leisure-time activity were estimated by multiplying the reported daily time spent in each activity by its corresponding MET intensity. Medical and medication histories were assessed using self-administered questionnaires. Hypertension, dyslipidemia (specifically hyperlipidemia), and diabetes were defined by the presence or absence of a medical history and/or current use of medication.

Body composition was examined using bioelectrical impedance analysis to measure skeletal muscle mass. An HBF-375 (Omron Healthcare Co., Ltd. Kyoto, Japan) was used to measure the body fat mass (BFM) and skeletal muscle mass (SMM) [22]. Both left and right grip strengths were measured once using a Smedley Hand Dynamometer (Matsumiya Medical Precision Machinery Co., Tokyo, Japan), and the maximum value was used for analysis.

### 2.4. Statistical Analysis

Continuous variables were expressed as mean ± standard deviation or range. Multiple regression analysis was performed to assess the combined influence of variables on serum BA levels. To account for effects of various factors on serum BA levels, we adjusted for the following variables for each serum BA: age, daily alcohol consumption, BMI, BFM, SMM, METs, hand grip strength, and dietary energy, fiber, fat, cholesterol, green tea, and coffee intake. In the comparison of quartile groups, one-way analysis of variance was conducted to assess the significance of the difference between groups. All statistical analyses were performed using SPSS statistical software version 25 (IBM Japan, Tokyo, Japan), and *p* < 0.05 was considered statistically significant.

## 3. Results

### 3.1. Association of BA Profile with Dietary Habits

**Figure 1:**
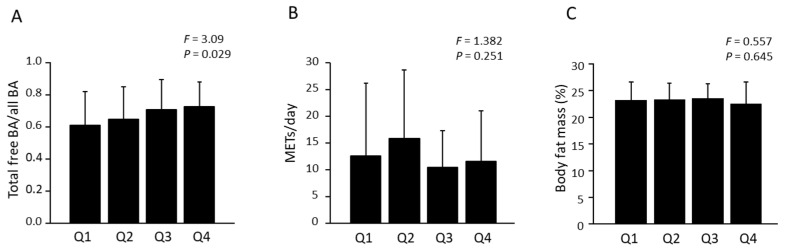
Comparison of deconjugation ratio of serum BA, physical activity, and body fat in quartile groups of green tea intake. Total free BA/All BA ratio (**A**), METs (**B**), and body fat (**C**) in quartile groups. Values are expressed as mean ± standard deviation. METs: total METs (>3 METs) multiplied by hour. Q1: minimum intake group, Q4: maximum intake group.

Green tea intake was negatively associated with the serum levels of secondary BAs, free DCA (*p* = 0.078), glyco-DCA (*p* = 0.048), and tauro-DCA (*p* = 0.037) (Table 1) and was positively associated with the deconjugation ratio of BA (the total free BAs/all BAs ratio) (*p* = 0.028). In contrast to the negative association between green tea consumption and secondary BAs and the positive association with deconjugation ratios, no similar associations were observed with coffee consumption. In the quartile comparison in green tea consumption, the deconjugation ratio showed a significant difference (*p* = 0.029); the higher green tea intake group showed a higher deconjugation ratio (Figure 1A). In contrast, body fat and METs, typical factors that can influence metabolic capacity and microbiota in green tea studies [23,24,25], showed no significant differences between quartile groups (Figure 1B,C). Intake of energy, fat, and cholesterol intake did not show any association with deconjugation ratio and secondary BAs except for tauro-UDCA. Dietary fiber intake showed positive correlations with the levels of secondary BAs, free DCA (*p* = 0.029), free LCA (*p* = 0.030), glyco-DCA (*p* = 0.010), and tauro-DCA (*p* = 0.019).

### 3.2. Association of BA Profile with Fitness Parameters and Physical Activity

METs were negatively associated with the levels of secondary BAs, free DCA (*p* = 0.098), and free LCA (*p* = 0.009) (Table 2). In assessments of primary BAs, METs were positively associated with the levels of conjugated BAs, total primary glyco-BA (*p* = 0.012), and total primary tauro-BA (*p* = 0.005). A negative association was found between METs and the deconjugation ratio (the total free BA/all BA ratio) (*p* = 0.029). Hand grip strength was negatively associated with tauro-DCA (*p* = 0.041), total free BA (*p* = 0.033), and all BA (*p* = 0.007) levels. It also showed positive associations with glyco-CA (*p* = 0.008) and glyco-CDCA (*p* = 0.023) levels. BFM and SMM showed positive trends with glyco-DCA (*p* = 0.099) and total free BA (*p* = 0.089) levels, respectively. 

## 4. Discussion

In this observational study, green tea consumption was positively associated with the deconjugation ratio of all BAs in peripheral blood. The higher quartile group of green tea consumption showed a higher ratio of BA deconjugation. In addition, green tea consumption, as well as METs and hand grip strength, were negatively associated with serum DCA levels. Based on BA characteristics, the associations of secondary BA levels and deconjugation ratio with green tea consumption, physical activity, and strength suggest that changes in BAs are involved in their systemic and intestinal beneficial modulations.

Several intestinal bacteria with bile salt hydrolase (BSH) activity mediate the deconjugation of conjugated BAs and convert them to free BAs [26]. Thus, the deconjugation ratio can indirectly indicate microbiota composition. Green tea contains polyphenol compounds, especially (−)-epigallocatechin-3-gallate (EGCG), (−)-epicatechin-3-gallate (ECG), (−)-epigallocatechin (EGC), and (−)-epicatechin (EC), which have antioxidant, anti-bacterial, and other specific effects [27]. In an in vitro study, tea catechins inhibited the growth of pathogens such as *Staphylococcus aureus*, *Escherichia coli* O157:H7, and *Salmonella typhimurium* DT104 [28] and other bacteria such as *Bacteroides-Prevotella*, *Clostridium histolyticum*, and *Eubacterium-Clostridium* group [29]. In contrast, the abundance of beneficial bacteria such as *Bifidobacterium* spp. and *Lactobacillus*/*Enterococcus* was increased by tea catechins, leading to the production of short-chain fatty acids [29]. Intake of green tea and catechins has been reported to modulate intestinal microbiota composition in humans and mice. In healthy humans, daily green tea consumption increases the proportion of *Bifidobacterium* and *Butyricimonas* [24,30]. Mice fed a high-fat diet supplemented with catechin for 4–8 weeks showed lower levels of Firmicutes, higher levels of Bacteroidetes, and higher diversity than those fed a normal diet [31,32]. Some of the changed bacteria have high BSH activity, which may contribute to the accelerated deconjugation of BA by green tea consumption. Indeed, in the animal study, although the deconjugation ratio of primary BAs decreased in the HFD-fed condition, it was restored by catechin supplementation [32]. Thus, our findings for BA deconjugation may be associated with microbiota modifications induced by green tea consumption. Physical activity and body fat are known factors that can influence microbiota [33,34]. However, we observed a higher ratio of BA deconjugation in the higher quartile group of green tea consumption while no significant changes were observed in physical activity and body fat between the quartile groups. This finding supports our hypothesis that green tea consumption modulates BA deconjugation and microbiota independently of physical activity and body fat.

Deconjugation of several BAs can modulate metabolic and inflammatory conditions. Generally, circulating BAs act as the specific receptor-mediated signaling factor [35]. Furthermore, their effects are not necessarily limited to those routes; the ability to deconjugate also contributes to metabolic improvement via attenuated inflammatory responses in skeletal muscle and adipocytes [11,12]. In contrast, conjugated BAs can directly disrupt the plasma membrane and activate the protein kinase C pathway, resulting in inflammatory responses [36,37]. Such BA-induced effects may mediate metabolic and inflammation improvement by green tea consumption, as shown in previous studies [38,39]. Green tea intake has been shown to improve glucose and lipid metabolism and exhibit anti-inflammatory effects [38,40]. Especially, catechins, a major ingredient, can directly affect metabolic organs such as skeletal muscle and adipose tissue, and accelerate glucose uptake and lipolysis [41,42]. Our observation suggests that green tea may exert not only direct effects but also indirect effects through BA modulation.

Deconjugation of primary BAs has been shown to be accelerated in the feces of exercised mice [11]. Transplantation of fecal microbiota from donor mice acclimated to running exercise has been shown to improve glucose tolerance with high BA deconjugation under a high-fat diet. Free BA levels show a greater glucose metabolic effect than conjugated BAs. Human studies have also shown that daily exercise increases the proportion of microbiota with BSH activity [43]. However, this study did not find a positive correlation between physical activity and the deconjugation ratio of primary BAs. Several possible explanations can be provided for these contradictory results. First, the beneficial effects of exercise are not only mediated by intestinal microbiota. In addition to the composition of microbiota, exercise also causes changes in hormones, cytokines, and metabolites and activates various physiological functions, which may counteract the deconjugating effects of intestinal microbiota. Second, the responses of various functions, such as muscle contraction, respiratory circulation, and hormone secretion, differ between exercise training and leisure-time physical activity in daily life. The adaptation of energy metabolism and vascular functions to exercise depends on the exercise intensity. In the previously reported animal study [11], the mice performed running exercises that gradually increased in time and speed over 4 weeks to relatively high-intensity, which was designed to induce efficient adaptation. Therefore, the effects on the intestinal microbiota and BAs may depend on the intensity and duration of exercise. Third, the results of the animal studies were obtained under high-fat diet conditions [11]. High-fat diets disrupt intestinal microbiota and weaken deconjugation [15,32]. Under these conditions, the exercise intervention may have had an effect. Finally, the fecal transit time in the gastrointestinal tract may have influenced the findings. Exercise is known to shorten fecal transit time and improve constipation [44], which may prevent the absorption of BAs into the circulation. Given the negative correlations of METs with free secondary BAs and their positive correlations with primary conjugated BAs, physical activity may accelerate BA synthesis and induce increased primary conjugated BA levels as well as decreased secondary free BA levels, which may lead to low deconjugation ratios of total BAs. In high-fat-fed rodents, five weeks of running exercise was shown to accelerate the BA synthesis pathway in the liver [45]. A 12-week voluntary running was also shown to accelerate BA synthesis in the hypercholesterolemic condition [46]. Further research is required to clarify the relationship between physical activity and BA deconjugation. 

The stool secondary BA profile has been shown to be involved in the risk of intestinal diseases associated with lifestyle factors such as diet and physical activity. A meta-analysis showed that stool DCA levels were positively associated with the incidence of colon cancer [47]. Secondary BAs inhibit the proliferation of bacteria such as *Lactobacillus* and *Bifidobacterium*, which are beneficial to the host. Abnormal increases in the DCA and LCA levels cause oxidative stress, apoptosis, and DNA damage, which can trigger colon tumorigenesis [48]. Typically, high DCA and LCA levels in high-fat diets can cause diet-induced colon tumorigenesis [49]. Several epidemiological studies have demonstrated that physical activity and green tea consumption are typical lifestyle factors that reduce the risk of colon tumorigenesis [50,51]. In animal studies, exercise and green tea interventions have been shown to reduce fecal secondary BA levels, which supports their potential role in preventing colon tumorigenesis. A recent study suggested that the circulating BA profile is more suitable for screening colorectal cancer than stool BA levels [52], which supports the significance of serum BA profiles. In addition, secondary BAs can cause abnormal intestinal permeability and allow the invasion of extrinsic factors into the bloodstream [13,14], leading to systemic low-grade inflammation and metabolic dysfunction. Thus, the negative association of serum secondary BAs with green tea consumption, as well as physical activity and strength, may indicate the reduced risk of colon tumorigenesis and leaky gut conditions by those lifestyles. Considering the process of intestinal BA metabolism, an accelerated deconjugation step of primary BAs generally leads to increased subsequent conversion steps to secondary BAs. Nevertheless, green tea intake showed both a positive association with deconjugation ratios and a negative association with secondary BAs. Further studies to elucidate such effects are indicated. In contrast, a positive correlation was observed between dietary fiber intake and secondary BAs. Although this cross-sectional study does not reveal a causal relationship between those factors, it is challenging to conclude that dietary fiber intake increases secondary BAs, considering several basic experiments and intervention studies. One possible reason is that the dietary survey based on the FFQ provides limited details about the specific foods containing dietary fiber. Consequently, many confounding factors with other ingredients in the foods may be involved. Moreover, different types of dietary fiber can have different actions on the intestinal environment [53]. In the future, the different effects of food sources and the type of dietary fiber should be examined.

One limitation of this study is that the broad scope of the study necessitated the examination of numerous variables. Thus, further detailed analyses factorizing these variables are required to grade the influential factors for each BA. Furthermore, although the causal relationships between associated factors were unclear in this observation study, intervention studies are required to clarify the effect of lifestyle on the BA profile. 

## 5. Conclusions

Herein, we demonstrated an association between lifestyle and circulating BA composition. This study primarily explored associations of dietary habits, physical activity habits, and physical fitness with the circulating BA profile. Based on the results of the serum BA profile, diet, physical activity, and fitness in healthy middle-aged adults, we found that the deconjugation ratio of total BA was positively associated with green tea consumption. In addition, green tea intake, physical activity, and strength were negatively associated with serum DCA levels. These changes in the BA profile may be related to the health benefits of such a lifestyle, which are associated with gut microbiota modification. 

Considering the influence of intestinal microbiota on circulating BAs, a signal transducer, future studies should focus on targeting the microbes involved in BA metabolism from a research perspective. Furthermore, the findings of this study can be extended to elucidate novel mechanisms underlying the functions of food items that promote metabolic improvement. Several polyphenols such as catechins, quercetin, and anthocyanins are known to improve metabolic activity and biogenesis of mitochondria [3,4]; however, they are poorly absorbed in the intestinal tract. Therefore, indirect effects exerted via the gut microbiota-induced BA modification may partly improve the metabolic activity.

## Figures and Tables

**Table 1:** Association of serum bile acid levels with dietary habits.

		Energy Intake		Fat Intake		Cholesterol Intake		Dietary Fiber Intake		Green Tea Intake		Coffee Intake	
Dependent Variables		β	*p*-Value	β	*p*-Value	β	*p*-Value	β	*p*-Value	β	*p*-Value	β	*p*-Value
Free form													
	Free CA	0.045	0.651	0.005	0.970	−0.065	0.534	−0.019	0.862	0.067	0.450	−0.212	0.015
	Free CDCA	0.180	0.071	−0.003	0.980	−0.087	0.407	−0.125	0.264	0.025	0.778	−0.191	0.028
	Free DCA	−0.060	0.531	−0.007	0.959	−0.068	0.502	0.240	0.029	−0.152	0.078	0.134	0.112
	Free LCA	−0.096	0.326	−0.045	0.728	−0.033	0.751	0.241	0.030	−0.091	0.296	0.104	0.221
	Free UDCA	0.143	0.143	−0.165	0.200	0.149	0.149	−0.083	0.449	−0.074	0.391	−0.100	0.240
	Total primary free BAs	0.132	0.185	0	0.999	−0.082	0.437	−0.087	0.437	0.043	0.626	−0.207	0.018
	Total free BAs	0.172	0.083	−0.061	0.638	0.083	0.429	−0.066	0.555	−0.064	0.466	−0.146	0.093
Conjugated form													
	Glyco-CA	−0.073	0.447	−0.225	0.075	0.202	0.047	0.019	0.863	−0.025	0.769	−0.041	0.624
	Glyco-CDCA	−0.132	0.174	−0.101	0.428	0.089	0.386	−0.058	0.593	−0.062	0.469	−0.081	0.338
	Glyco-DCA	−0.114	0.237	−0.06	0.633	−0.129	0.206	0.285	0.010	−0.170	0.048	0.129	0.125
	Glyco-LCA	−0.137	0.164	0.145	0.260	−0.060	0.564	0.154	0.165	−0.135	0.124	0.075	0.379
	Glyco-UDCA	0.067	0.486	−0.168	0.188	0.160	0.120	−0.069	0.530	−0.089	0.301	−0.092	0.278
	Total primary glyco-BA	−0.118	0.219	−0.147	0.245	0.131	0.198	−0.035	0.744	−0.053	0.537	−0.071	0.395
	Total glyco-BA	−0.173	0.074	−0.055	0.665	−0.073	0.472	0.045	0.676	−0.187	0.030	0.101	0.229
	Tauro-CA	−0.005	0.954	−0.203	0.103	0.263	0.009	−0.025	0.816	−0.062	0.463	−0.066	0.426
	Tauro-CDCA	−0.11	0.233	−0.212	0.081	0.276	0.005	−0.087	0.404	−0.067	0.415	−0.15	0.063
	Tauro-DCA	−0.185	0.051	−0.053	0.666	−0.036	0.720	0.252	0.019	−0.175	0.037	0.179	0.031
	Tauro-LCA	−0.039	0.694	0.085	0.521	0.153	0.150	−0.114	0.317	−0.006	0.943	0.096	0.274
	Tauro-UDCA	0.082	0.398	−0.18	0.157	0.212	0.039	−0.088	0.421	−0.063	0.461	−0.064	0.447
	Total primary tauro-BA	−0.094	0.306	−0.217	0.074	0.282	0.004	−0.078	0.452	−0.068	0.407	−0.140	0.084
	Total tauro-BA	−0.150	0.117	−0.265	0.037	0.144	0.156	0.121	0.263	−0.138	0.107	0.059	0.482
All BA		0.062	0.523	−0.158	0.217	0.150	0.146	−0.054	0.620	−0.100	0.248	−0.129	0.129
Total free BA/All BA		0.177	0.067	0.075	0.551	0.057	0.577	−0.054	0.621	0.189	0.028	−0.101	0.229
Total glyco-BA/Total tauro-BA		0.052	0.606	0.073	0.583	−0.071	0.506	0.026	0.818	−0.040	0.661	−0.123	0.164
Total primary BA/All primary BA		0.168	0.089	−0.028	0.829	0.066	0.522	−0.017	0.877	0.141	0.107	−0.148	0.084

Adjusted for age, daily alcohol consumption, BMI, BFM, SMM, METs, hand grip strength, and dietary energy, fiber, fat, cholesterol, green tea, and coffee intake. CA, cholic acid; CDCA, chenodeoxycholic acid; DCA, deoxycholic acid; LCA, lithocholic acid; UDCA, ursodeoxycholic acid; BA, bile acid.

**Table 2:** Association of serum bile acid levels with indicators of body composition, physical activity, and strength.

		BFM		SMM		METs		Grip Strength	
Dependent Variables		β	*p*-Value	Β	*p*-Value	β	*p*-Value	β	*p*-Value
Free form									
	Free CA	−0.048	0.754	0.079	0.516	−0.043	0.634	−0.076	0.394
	Free CDCA	0.021	0.750	0.030	0.334	0.004	0.723	0.005	0.694
	Free DCA	0.014	0.270	0.020	0.284	−0.145	0.098	0.004	0.880
	Free LCA	0.085	0.570	0.088	0.464	−0.234	0.009	−0.103	0.245
	Free UDCA	0.180	0.231	0.162	0.179	−0.026	0.772	−0.245	0.006
	Total primary free BA	−0.050	0.742	0.107	0.382	−0.038	0.676	−0.054	0.551
	Total free BA	0.157	0.304	0.208	0.089	−0.083	0.358	−0.193	0.033
Conjugated form									
	Glyco-CA	0.009	0.941	0.191	0.195	0.043	0.718	0.233	0.008
	Glyco-CDCA	0.051	0.664	0.098	0.509	0.051	0.667	0.201	0.023
	Glyco-DCA	0.245	0.099	0.147	0.217	−0.012	0.892	−0.036	0.681
	Glyco-LCA	−0.085	0.575	−0.100	0.406	−0.078	0.380	−0.080	0.368
	Glyco-UDCA	0.174	0.245	0.142	0.234	0.003	0.975	−0.253	0.004
	Total primary glyco-BA	0.134	0.365	0.051	0.667	0.221	0.012	−0.102	0.241
	Total glyco-BA	0.104	0.483	0.031	0.793	0.193	0.028	−0.035	0.687
	Tauro-CA	0.066	0.647	0.013	0.912	0.290	0.001	−0.091	0.287
	Tauro-CDCA	0.035	0.807	0.016	0.888	0.215	0.011	−0.174	0.038
	Tauro-DCA	−0.014	0.925	−0.102	0.378	−0.123	0.150	−0.175	0.041
	Tauro-LCA	−0.076	0.625	0.024	0.848	−0.143	0.118	0.048	0.595
	Tauro-UDCA	0.143	0.337	0.138	0.246	0.020	0.819	−0.248	0.005
	Total primary tauro-BA	0.041	0.770	0.016	0.888	0.235	0.005	−0.165	0.050
	Total tauro-BA	0.108	0.462	−0.031	0.790	0.105	0.227	−0.092	0.291
All BA		0.211	0.159	0.189	0.115	0.041	0.641	−0.240	0.007
Total free BA/All BA		−0.108	0.466	−0.027	0.822	−0.192	0.029	0.041	0.635
Total glyco-BA/Total tauro-BA		0.132	0.396	0.149	0.234	0.113	0.220	−0.022	0.806
Total primary BA/All primary BA		−0.070	0.642	0.062	0.606	−0.121	0.174	0.072	0.420

Adjusted for age, daily alcohol consumption, BMI, BFM, SMM, METs, hand grip strength, and dietary energy, fiber, fat, cholesterol, green tea, and coffee intake. BFM, body fat mass; SMM, skeletal muscle mass; MET, metabolic equivalent; CA, cholic acid; CDCA, chenodeoxycholic acid; DCA, deoxycholic acid; LCA, lithocholic acid; UDCA, ursodeoxycholic acid; BA, bile acid.

## Data Availability

The datasets generated during and/or analyzed during the current study are available from the corresponding author on reasonable request.

## Supplementary Materials

The following supporting information can be downloaded at: https://www.mdpi.com/article/10.3390/nu16193381/s1, Figure S1: Flow chart of the study participants; Table S1: Negative ESI-SRM and HPLC data of each bile acid.
