# Supplementary material for: Association of Serum Bile Acid Profile with Diet and Physical Activity Habits in Japanese Middle-Aged Men
Source: Nutrients. 2024 Oct 4;16(19):3381. doi: 10.3390/nu16193381 (PMC11478694; doi:10.3390/nu16193381)
Supplement: Supplementary file 1 [file nutrients-16-03381-s001.zip › Nutrients 2024_Suppl Table.pdf]

Table S1. Negative ESI-SRM and HPLC data of each bile acid.

ID	Bile acids	SRM Condition		HPLC Data (RRT[a])	Internal Standards for Quantification[b]
		Precursor to Product (*m/z*)	Collision Energy (*V*)		
1	CA	407 → 407	20	1.00	A
2	GCA	464 → 74	35	0.93	A
3	TCA	514 → 80	70	0.95	F
4	CDCA	391 → 391	20	1.26	B
5	GCDCA	448 → 74	35	1.14	B
6	TCDCA	498 → 80	70	1.16	G
7	DCA	391 → 391	20	1.30	C
8	GDCA	448 → 74	35	1.19	C
9	TDCA	498 → 80	70	1.21	G
10	LCA	375 → 375	20	1.57	D
11	GLCA	432 → 74	35	1.39	D
12	TLCA	482 → 80	70	1.39	G
13	UDCA	391 → 391	20	0.92	E
14	GUDCA	448 → 74	35	0.83	E
15	TUDCA	498 → 80	70	0.85	G
**Internal standards**					
A	[2,2,4,4-$^{2}H_{4}$]CA	411 → 411	20	1.00	—
B	[11,11,12,12-$^{2}H_{4}$]CDCA	395 → 395	20	1.26	—
C	[2,2,4,4-$^{2}H_{4}$]DCA	395 → 395	20	1.30	—
D	[2,2,4,4-$^{2}H_{4}$]LCA	379 → 379	20	1.57	—
E	[11,11,12,12-$^{2}H_{4}$]UDCA	395 → 395	20	0.92	—
F	[2,2,3,4,4-$^{2}H_{5}$]TCA	519 → 80	70	0.95	—
G	[11,11,12,12-$^{2}H_{4}$]TUDCA	502 → 80	70	0.85	—

Abbreviations: ESI, electrospray ionization; SRM, selected reaction monitoring; RRT, relative retention time.

[a]RRTs are expressed relative to the retention time of CA. A reversed phase C18 column, Hypersil GOLD (150 mm x 2.1 mm I.D., 3 μm, Thermo Fisher Scientific) was employed. Initially, the mobile phase was comprised of 20 mM ammonium acetate buffer (pH 7.5)–acetonitrile–methanol (70:15:15, v/v/v), then it was programmed in a linear manner to a 30:35:35 (v/v/v) over 20 min. The final mobile phase was kept constant for an additional 10 min. The flow rate was 200 μl/min, and the column was maintained at 40°C using a column oven. The retention time of CA by this condition was around 16.4 min.

[b]ID of internal standard used for the quantification of each bile acid (BA). Calibration curves for BAs were established each time in the analysis. Different amounts of authentic BAs were mixed with deuterated internal standards and quantified for calibration curves. The weight ratio of each BA, relative to the corresponding deuterated internal standard, was plotted on the abscissa, and the peak area ratio of the authentic BA to the deuterated variant measured by SRM was plotted on the ordinate.
