# Supplementary figures and images for: Association of Serum Bile Acid Profile with Diet and Physical Activity Habits in Japanese Middle-Aged Men
Source: Nutrients. 2024 Oct 4;16(19):3381. doi: 10.3390/nu16193381 (PMC11478694; doi:10.3390/nu16193381)

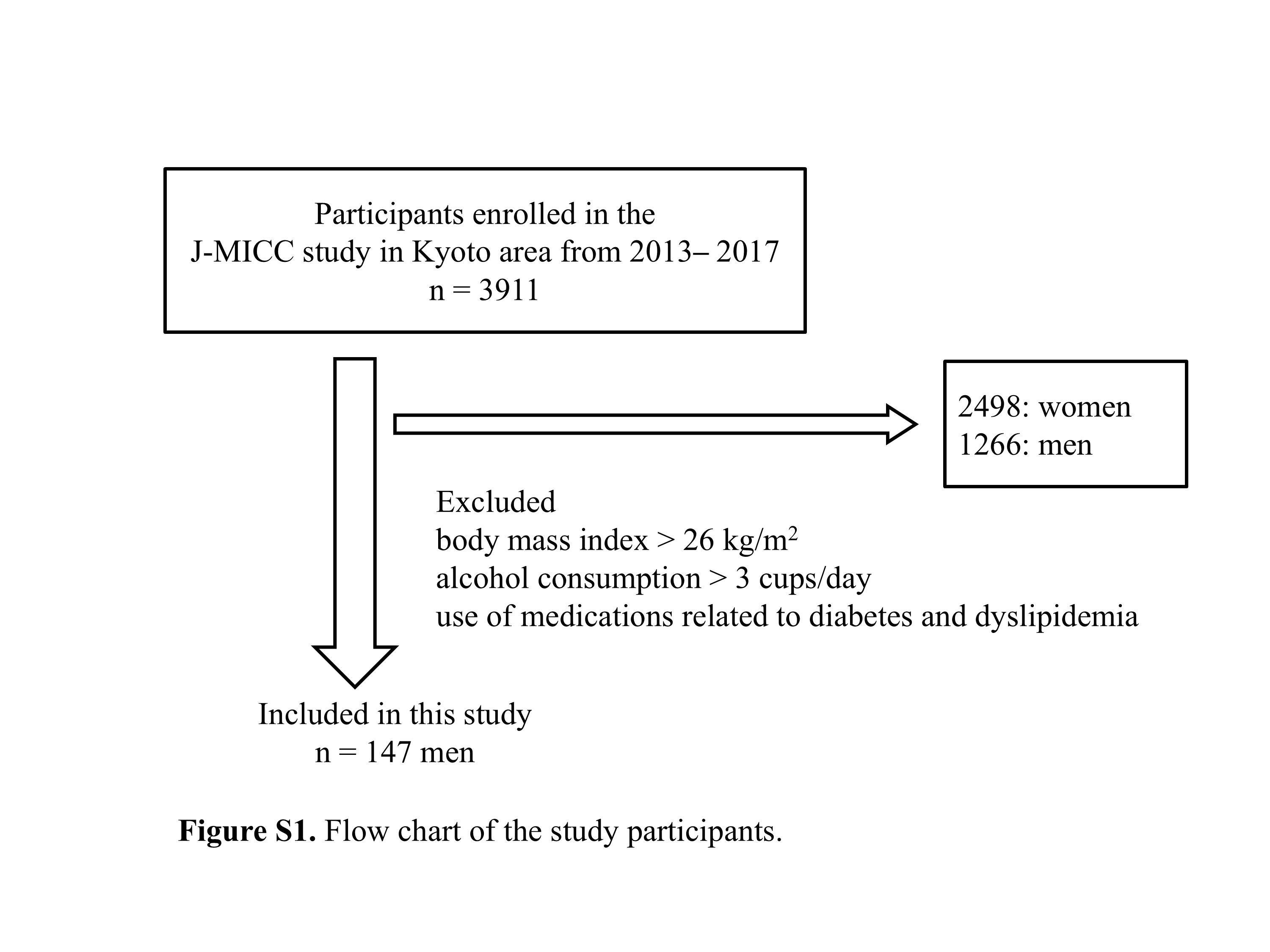

Supplement: Supplementary file 1 [file nutrients-16-03381-s001.zip › Nutrients 2024_Suppl Fig.tif]
